# The role of human umbilical cord tissue-derived mesenchymal stromal cells (UCX®) in the treatment of inflammatory arthritis

**DOI:** 10.1186/1479-5876-11-18

**Published:** 2013-01-17

**Authors:** Jorge M Santos, Rita N Bárcia, Sandra I Simões, Manuela M Gaspar, Susana Calado, Ana Água-Doce, Sílvia CP Almeida, Joana Almeida, Mariana Filipe, Mariana Teixeira, José P Martins, Luís Graça, Maria EM Cruz, Pedro Cruz, Helder Cruz

**Affiliations:** 1 ECBio – Investigação e Desenvolvimento em Biotecnologia, S.A., R. Henrique Paiva Couceiro,27, Amadora, 2700-451, Portugal; 2 Research Institute for Medicines and Pharmaceutical Sciences (iMed.UL), Faculdade de Farmácia, Universidade de Lisboa, Av. Prof. Gama Pinto, Lisboa, 1649-003, Portugal; 3 Instituto de Medicina Molecular, Faculdade de Medicina da Universidade de Lisboa, Av. Professor Egas Moniz, Lisboa, 1649-028, Portugal

**Keywords:** UCX® cells, Umbilical cord tissue, Mesenchymal stromal cells, Anti-inflammatory, Immunosuppressive, Autoimmune, Arthritic inflammation

## Abstract

**Background:**

ECBio has developed proprietary technology to consistently isolate, expand and cryopreserve a well-characterized population of stromal cells from human umbilical cord tissue (UCX® cells). The technology has recently been optimized in order to become compliant with Advanced Medicine Therapeutic Products. In this work we report the immunosuppressive capacity of UCX® cells for treating induced autoimmune inflammatory arthritis.

**Methods:**

UCX® cells were isolated using a proprietary method (PCT/IB2008/054067) that yields a well-defined number of cells using a precise proportion between tissue digestion enzyme activity units, tissue mass, digestion solution volume and void volume. The procedure includes three recovery steps to avoid non-conformities related to cell recovery. UCX® surface markers were characterized by flow cytometry and UCX® capacity to expand *in vitro* and to differentiate into adipocyte, chondrocyte and osteoblast-like cells was evaluated. Mixed Lymphocyte Reaction (MLR) assays were performed to evaluate the effect of UCX® cells on T-cell activation and Treg conversion assays were also performed *in vitro.* Furthermore, UCX® cells were administered *in vivo* in both a rat acute carrageenan-induced arthritis model and rat chronic adjuvant induced arthritis model for arthritic inflammation. UCX® anti-inflammatory activity was then monitored over time.

**Results:**

UCX® cells stained positive for CD44, CD73, CD90 and CD105; and negative for CD14, CD19 CD31, CD34, CD45 and HLA-DR; and were capable to differentiate into adipocyte, chondrocyte and osteoblast-like cells. UCX® cells were shown to repress T-cell activation and promote the expansion of Tregs better than bone marrow mesenchymal stem cells (BM-MSCs). Accordingly, xenogeneic UCX® administration in an acute carrageenan-induced arthritis model showed that human UCX® cells can reduce paw edema in vivo more efficiently than BM-MSCs. Finally, in a chronic adjuvant induced arthritis model, animals treated with intra-articular (i.a.) and intra-peritoneal (i.p.) infusions of UCX® cells showed faster remission of local and systemic arthritic manifestations.

**Conclusion:**

The results suggest that UCX® cells may be an effective and promising new approach for treating both local and systemic manifestations of inflammatory arthritis.

## Background

Many forms of inflammatory arthritis are autoimmune disorders, in which the body identifies its own tissues as foreign and reacts with inflammation. Autoimmune conditions include Rheumatoid Arthritis, Lupus, Ankylosing Spondylitis, Reiter’s Syndrome, Psoriatic Arthritis and arthritis associated with Inflammatory Bowel Disease [1]. Rheumatoid arthritis (RA), for example, is an immunologically mediated chronic and systemic disease where synovial joints are attacked leading to articular destruction and functional disability. The molecular defect causing RA has not been characterized, but may involve aberrant T-cell, B cell, and macrophage function. Although RA often responds to immunosuppressive medication including corticosteroids, methotrexate, azathioprine and cyclophosphamide, or to non-steroidal anti-inflammatory drugs, no therapy has been curative and often patients become non-responsive to therapeutic drugs. More recent efforts to discover new target therapies have achieved some success in alleviating inflammation; for instance, TNF-α blockers, like the monoclonal antibodies etanercept (Enbrel), infliximab (Remicade), adalimumab (Humira), certolizumab pegol (Cimzia) and golimumab (Simponi), and B-Lymphocyte depleting therapies, like rituximab (Rituxan), have benefited many RA patients [2,3]. However, these approaches are very expensive and none of the currently widely used biological agents reaches long term drug-free remission [4,5]. It is therefore important to develop new and more effective therapies for autoimmune-based inflammatory arthritis.

Mesenchymal stromal/stem cells (MSCs) display immunosuppressive and anti-inflammatory properties, and their putative therapeutic role in a variety of inflammatory autoimmune diseases is currently under investigation [6-8]. An essential characteristic of MSCs is that they express a variety of chemokine and cytokine receptors that drive them to sites of inflammation [9-12]. Thus, the immune-regulatory effects of MSCs occur in a localized environment and are not systemic, unlike steroid therapy where systemic suppression can lead to major clinical complications [13]. MSCs derived from umbilical cord tissue (UC-MSCs) have been specifically shown to be viable for allogeneic applications due to both their low immunogenicity, even when compared to MSCs from other sources, and their capacity for localized immunosuppression. In fact, immunosuppression activity of UC-MSCs is thought to be enhanced by inflammation signals and can be induced in vitro by pro-inflammatory treatment with IFN-γ or TNF-α [14,15]. More recently, UC-MSCs have been shown to be capable of inhibiting proliferation of fibroblast-like synoviocytes (FLSs), inducing hyporesponsiveness of T-cells and promoting the expansion of regulatory T-cells (Tregs) from RA patients in vitro [12,16,17]. More importantly, systemic infusion of human UC-MSCs reduced the severity of collagen-induced arthritis (CIA) in mouse models, through a mechanism involving down regulation of pro-inflammatory cytokine and chemokine levels [TNF-α, IL-6, monocyte chemoattractant protein-1, D-dimer (D-D), antithrombin-III (AT-III), thrombomodulin (TM)] and up regulation of the anti-inflammatory cytokine IL-10, as measured in sera of UC-MSCs-treated mice [16,17]. Overall, these results suggest that UC-MSCs might be a therapeutic strategy in RA.

ECBio has developed proprietary technology to consistently isolate, expand, and cryopreserve a well-characterized population of human stem cells derived from the umbilical cord tissue (Wharton’s jelly) named herein as UCX® cells [18]. The method has been specifically designed for clinical use. Briefly, the main distinguishing differences between UCX® isolation technology and other UC-MSC isolation methods described previously are (1) the introduction of a three-stage strategy for cell recovery which makes the method 100% reliable, (2) peeling off the amniotic membrane which reduces the frequency of microbial contamination and augments the purity of the resulting UCX® population by eliminating epithelial progenitors, (3) absence of tissue incisions and/or tissue mincing/crushing steps that could hinder its application in GMP settings and jeopardize stem cell phenotype due to excessive mechanical manipulation [19-23], and (4) using an optimized ratio between tissue mass, digestion enzyme activity units, overall solution volume and void volume which allows for UCX® cell-specific release, leaving cord vessels intact and therefore reducing contamination with endothelial and sub-endothelial cells from umbilical arteries and vein. The overall avoidance of cell mechanical manipulation is one of the major goals of regulatory authorities, not only for phenotype maintenance but also because the lack of complex incision steps will improve reproducibility and subsequent risk assessment. More recently, the method has been adapted according to advanced therapy medicinal product (ATMP) standards, as defined by the guideline on the minimum quality data for certification of ATMP (EMA/CAT/486831/2008/corr, 2010). The aim now is to gather non-clinical evidence supporting the application of the resulting UCX® cell population for the treatment of inflammatory arthritis. In this study, UCX® cells were isolated and expanded up to passage 5 (P5), and characterized in terms of expression of cell-surface markers, tri-lineage differentiation, and the capacity to suppress the activation of T-cells and induce Tregs in vitro*.* Furthermore, UCX® cells were xenogeneically used in both acute carrageenan-induced arthritis (CarrIA) and chronic adjuvant-induced arthritis (AIA) models for arthritic inflammation, and their anti-inflammatory action monitored over time. The results suggest that the use of UCX® cells may be an effective new approach for treating both local and systemic manifestations of inflammatory arthritis. The results also show that UCX® cells are more promising therapeutic agents than bone marrow-derived mesenchymal stem cells (BM-MSCs).

## Methods

### Ethics and regulatory

This study was approved by the Ethics Committee at the Cascais Hospital Dr. José de Almeida, in the scope of a research protocol between ECBio – Research & Development in Biotechnology, S.A. and HPP Saúde – Parcerias Cascais, S.A. Umbilical cord donations (n = 8) proceeded with written informed consents according to Directive 2004/23/EC which sets the standards of quality and safety for the donation, procurement, testing, processing, preservation, storage and distribution of human tissues and cells. All the experimental procedures were carried out with the permission of the local laboratory animal research committees in accordance with internationally accepted guidelines, especially taking in consideration the 3Rs rule of - Replacement, Refinement and Reduction. All animals were obtained from Charles River Laboratories (Santa Perpetua de Mogoda, Spain) and kept under standard laboratory conditions. All animals were acclimatized before the experiments and housed in plastic cages under standard laboratory conditions, fed commercial chow and acidified drinking water *ad libitum*.

### UCX® isolation and cryopreservation

Human UCX® cells were isolated according to Santos *et al*. (2008). Briefly, fresh human umbilical cords were obtained after full-term natural births, transported to the laboratory facilities in a sterile container containing saline buffer, in a hermetically sealed sterile container, and processed within a period up to 48 h. Identical cord tissue sections were digested, using a precise ratio between tissue mass, tissue digestion enzyme activity units, digestion solution volume and void volume using collagenase (Sigma-Aldrich, Co.). The procedure includes three recovery phases in order to avoid non-conformities related to percentage cell recovery. In the first cell recovery phase, cells dissociated from the tissue are recovered by a static horizontal incubation. The remaining cells are incubated in a static monolayer culture using basal medium [α-MEM, 1 g/L glucose (Sigma-Aldrich, Co.); 2.2 g/L sodium bicarbonate (Sigma-Aldrich, Co.), supplemented with 2 mM L-glutamine (Sigma-Aldrich, Co) and 20% FBS (Gibco, Life Technologies)], at 37°C in 7% CO_2_ humidified atmosphere, with medium change every 72 h until they reached confluence. Confluent cultures were cryopreserved in 10% dimethylsulphoxide stock solution (DMSO; Sigma-Aldrich, Co.) and FBS, using control rate temperature decrease.

### UCX® expansion and characterization

Human cryopreserved UCX® cells were thawed and expanded to P5 in complete culture medium (α-MEM, 20% FBS, 1 g/L glucose, 2 mM glutamine) with medium change every 72 h. Cells were characterized by their capacity to adhere to plastic in standard MSCs culture conditions. Surface marker expression was analyzed by flow cytometry using a Gallios imaging flow cytometer (Beckman Coulter Inc., CA, USA). The antibodies used and their conjugates were: anti-human CD105 - PE (eBioscience) anti-human CD73 – APC, anti-human CD90 – PE, anti-human CD14 - PerCp/Cy5.5, anti-human CD45 – PerCp/Cy5.5, anti-human CD34 – FITC, anti-human CD19 pacific blue, anti-human HLA-DR Pacific blue, isotype Pacific blue IgG1, isotype Pacific blue IgG2a), isotype IgG1k PerCp/Cy5.5 ), isotype IgG2a PerCp/Cy5.5, isotype IgG1k - PE, isotype IgG1k APC and isotype IgG1k FITC (all from Biolegend, Inc.). For flow cytometry studies cells were incubated for 1 h at 4°C with the antibodies in 2% (w/v) bovine serum albumin solution (Sigma-Aldrich, Co.), centrifuged and washed with phosphate-buffered saline (Sigma-Aldrich, Co.). To induce adipogenic differentiation, cultured cells were incubated in adipogenic differentiation medium, for 3 days, consisting of α-MEM supplemented with 20% FBS, 2 mM L-glutamine, 10 μg/mL insulin (Sigma-Aldrich, Co.), 200 μM indomethacin (Sigma-Aldrich, Co.), 0.5 mM isobutylmetylxantine (Sigma-Aldrich, Co.), and 1 μM dexamethasone (Sigma-Aldrich, Co.); and subsequently 1 day in adipogenic maintenance medium, consisting of α-MEM supplemented with 20% FBS, 2 mM L-glutamine and 10 μg/mL insulin. Medium replacement cycles were repeated during 21 days after which histochemical staining was performed. For chondrogenic differentiation, cells were grown in suspension as pellets, incubated with chondrogenic differentiation medium consisting on DMEM-LG (Sigma-Aldrich, Co.), 1% FBS, 2 mM L-glutamine, 6.25 μg/mL insulin (Sigma-Aldrich, Co.), 10 ng/mL TGF-β1 (Tebu-bio), and 50 μM ascorbate-2-phosphate (Sigma-Aldrich, Co.). The medium was replaced every 3 days during 21 days and histochemical staining was performed. Finally, to induce osteogenic differentiation, cells were incubated in osteogenic differentiation medium [α-MEM, 10% FBS, 1 g/L glucose, 2 mM glutamine, 10 mM β-glycerol phosphate, 50 μg/mL ascorbate-2-phosphate and 100 nM dexamethasone (all from Sigma-Aldrich, Co.)]. The medium was replaced every 3 days during 21 days and histochemical staining was performed. In adipogenic and osteogenic differentiation protocols, cells were washed and fixed with paraformaldehyde 4% for 20 min and stained with oil red O and alkaline phosphatase, respectively. For chondrogenic differentiation, cells were also fixed in paraformaldehyde 4%, dried and cut into sections and finally stained with alcian blue. The presence of stained cells was confirmed by inverted microscopy with phase contrast (Leica, DMIL HC).

### Mixed lymphocyte reaction (MLR)

Peripheral blood samples were obtained from healthy volunteers of both sexes after informed consent. The MLR was performed in 96-well microtiter plates using RPMI (Gibco), and 5% human serum obtained from the specific donor. Peripheral blood monocytes (PBMCs) were obtained from two different donors and cultured at 2 x 10^5^ cells per well. Stimulator cells were irradiated with 50 Gy (Gammacell ELAN 3000, Best Theratronics) prior to addition to the culture at 20,000 cells per well. Quadruplicate cultures were performed for each condition. Cultures were incubated at 37°C in 5% CO_2_ for 6 days, pulsed with [^3^H]thymidine (1 microCi per well, Amersham Biosciences, Piscataway) for 16 hours, and the cells were harvested onto filter mats using a Tomtec 96-well cell harvester (Perkin Elmer). Radioactivity incorporated into the dividing cells was determined using a scintillation counter (Microbeta Trilux Scintillation and Luminescence Counter 145 LSC, Perkin Elmer). BM-MSCs used as controls in this and following experiments were purchased from Innoprot (Cat. P10576) and grown according to supplier's up to P5.

### Cell sorting of CD3^+^CD4^+^CD25^-^ T-cells and cultures for conversion into CD4^+^CD25^+^Foxp3^+^ Tregs

Peripheral blood from healthy volunteers was collected in heparin, diluted 1:1 (v/v) with PBS and mixed with half the volume of Histopaque®-1077 (Sigma). Cells were collected from the Ficoll gradient after centrifugation at 720 *g* for 30’ at RT, washed with PBS containing 2% FCS and then stained with mAbs against human CD3, CD4 and CD25 (Ebioscience) for cell sorting. The purified CD3^+^CD4^+^CD25^-^ T-cells were cultured in plate-bound αhuCD3 (2.5 μg/ml, Ebioscience) in 96-well flat-bottom plates in the following conditions. Briefly, 1x10^5^ purified T-cells/well were cultured in the presence of αhuCD28 (2 μg/ml, Ebioscience), huIL-2 (20 U/ml, Peprotech), and TGF-β (10 ng/ml, R&D Systems) or the indicated cell lines (irradiated as described), in replacement of TGF-β, in a ratio of 1:1 to the T-cells. All conditions were performed in triplicate wells. After 5 days in culture at 37°C with 5% CO_2_, cells were stained with mAbs against human CD3, CD4 and CD25 (Ebioscience) and then stained for huFoxp3 as described by the manufacturer (Ebioscience). The analysis was performed on the converted CD4^+^Foxp3^+^ regulatory T-cells.

### Acute carragenan-induced arthritic (CarrIA) inflammatory model

Carrageenan and indomethacin were purchased from Sigma Aldrich (St. Louis, MO, USA). At least 6 male Wistar rats, minimum 7 to 8 weeks-old, were used per experimental group. Paw edema was induced by intradermal injection of 0.1 mL of a 1% carrageenan saline solution into the subplantar area of the right hind paw [24]. The evaluation of the paw edema was monitored by changes of the volume of right and left paws by a water displacement method, using a plethysmometer (Ugo Basile, Comerio, Italy). The paws were immersed in the measurement cell up to the hair line of the ankle to determine the immersed organ volume in mL. Measurements were made immediately before the injection of carrageenan and thereafter at 2-hr intervals for 6 hr. Edema was expressed as the increase in paw volume (milliliters) after carrageenan injection relative to the pre-injection value for each animal. Sub-cutaneous indomethacin (30 mg/kg) administration was performed 30 min before carrageenan injection. Cells at a concentration of 1.7 x10^6^ in a total volume of 0.1 mL or vehicle PBS (0.1 mL) were administered by intra articular (i.a.) injection in the right paw, 30 min before carrageenan injection.

### Chronic adjuvant-induced arthritis (AIA) model

*Mycobacterium butyricum* (killed and dried) and incomplete Freund´s Adjuvant were purchased from Difco Laboratoires, Detroit, Michigan, USA. All other chemicals were of analytical grade. Six to 8 male Wistar rats aged four months were used. As a rule, inflammation was induced by a single intra-dermal injection of 0.10 mL of a 10 mg/mL suspension of *M. butyricum* in Incomplete Freund's Adjuvant, homogenized by ultra-sound into the sub-plantar area of the right hind paws [25,26]. Before treatment, animals were randomly allocated to 6 different treatment groups. One group of induced animals remained untreated, being further referred as Control Untreated. Other 2 groups of induced animals received PBS by intra-articular (i.a.) or intra-peritoneal (i.p.) routes of administration, respectively; being further referred as Sham groups. These groups were kept under the same conditions as treated animals. A total of five i.p. administrations (dose: 1.000x10^6^ cells/injection) were performed every day, from day 13 to 17. In turn, three i.a. administrations (low dose: 0.425x10^6^ cells/injection; high dose: 1.700x10^6^ cells/injection) were performed every other day, for five days, from day 13 to 17. The experiment lasted for 55 days. The therapeutic effect of UCX® cells was evaluated by physical changes in several parameters: body weight, ankle circumference and volume of right and left paws. Animals were scored for clinical arthritis as previously described, with some modifications [27]. At pre-selected times animals were blindly graded based on the sum of the following grades: 0 = normal; 1 = for each inflamed paw; 1 = tail lesion; 1 = joint rigidity or deformity; 1 = wounded paw; 1 = infected paw; 1 = necrotic paw. The sum of the parameters was calculated as an arthritic index with a maximum possible score of 9. The evolution of clinical signs was photographically recorded and compared to naive animals. Treatment was initiated after the onset of the disease (day 13), when arthritis had become established (AI score of 4).

## Results

### UCX® cell characterization: cell culture and flow cytometry analysis of cell

#### Surface markers

**Figure 1:**
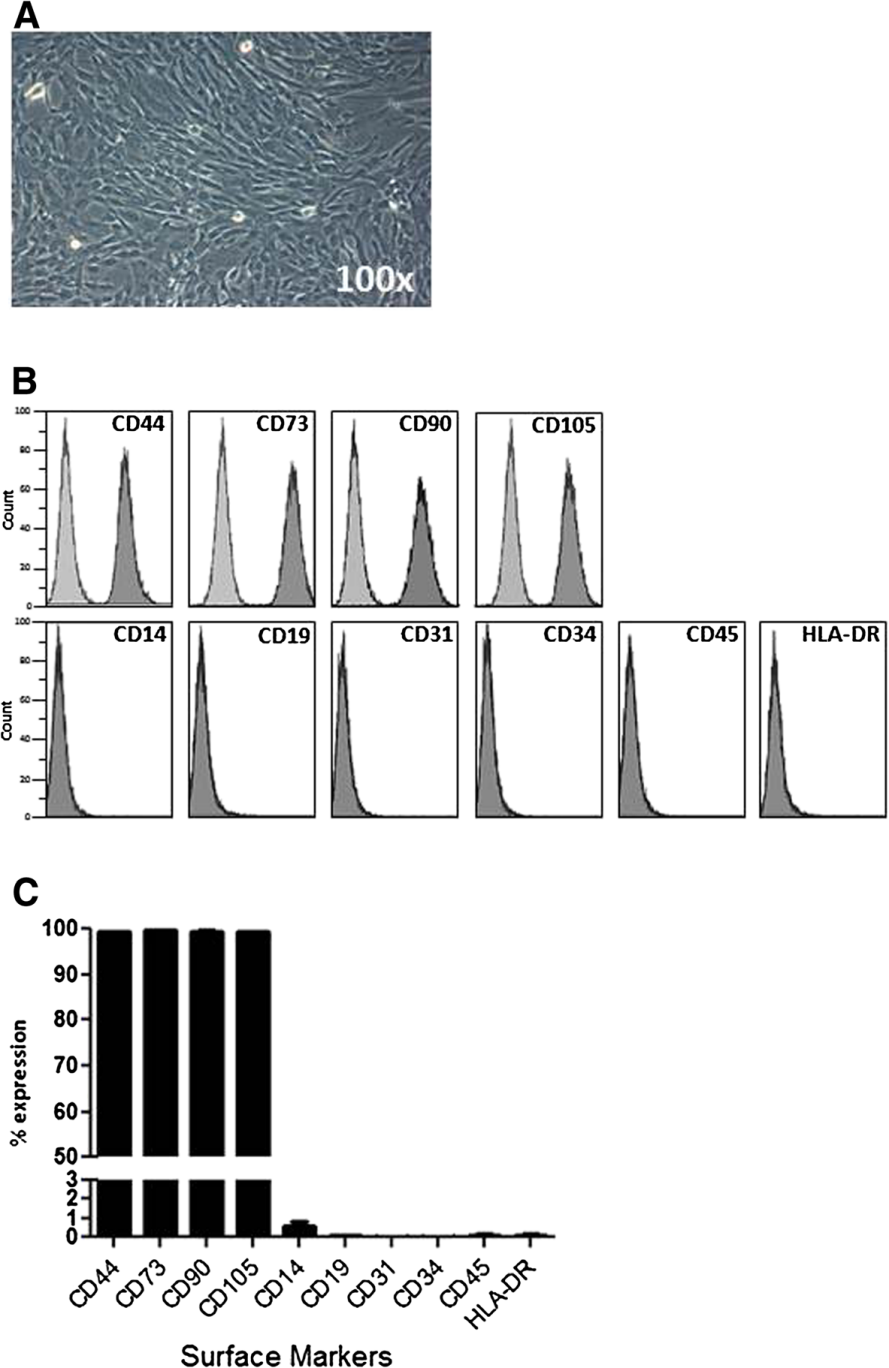
**UCX® cell characterization.** Isolated UCX® cells presented (**A**) a fusiform, fibroblast-like morphology in culture. Results from Flow Cytometry analysis of cell surface markers presented in (**B**) histograms (UCX® cells in dark grey and isotype control in light grey) and (**C**) column bars. The values are represented as the mean ± SEM (n = 8).

UCX® cells were expanded to P5 (12.3 ± 2.5 generations, n = 8) where the culture appeared homogeneous and cells presented their typical fusiform, fibroblast-like, morphology (Figure 1A). As expected for MSC-type stromal cells, flow cytometry analysis showed that over 98% of the cells in the population were consistently positive for the cell surface markers CD44, CD73, CD90 and CD105 and less than 2% positive for CD14, CD19, CD31, CD34, CD45 and HLA-DR (Figure 1B and 1C).

#### UCX® cell characterization: Tri-lineage terminal differentiation

**Figure 2:**
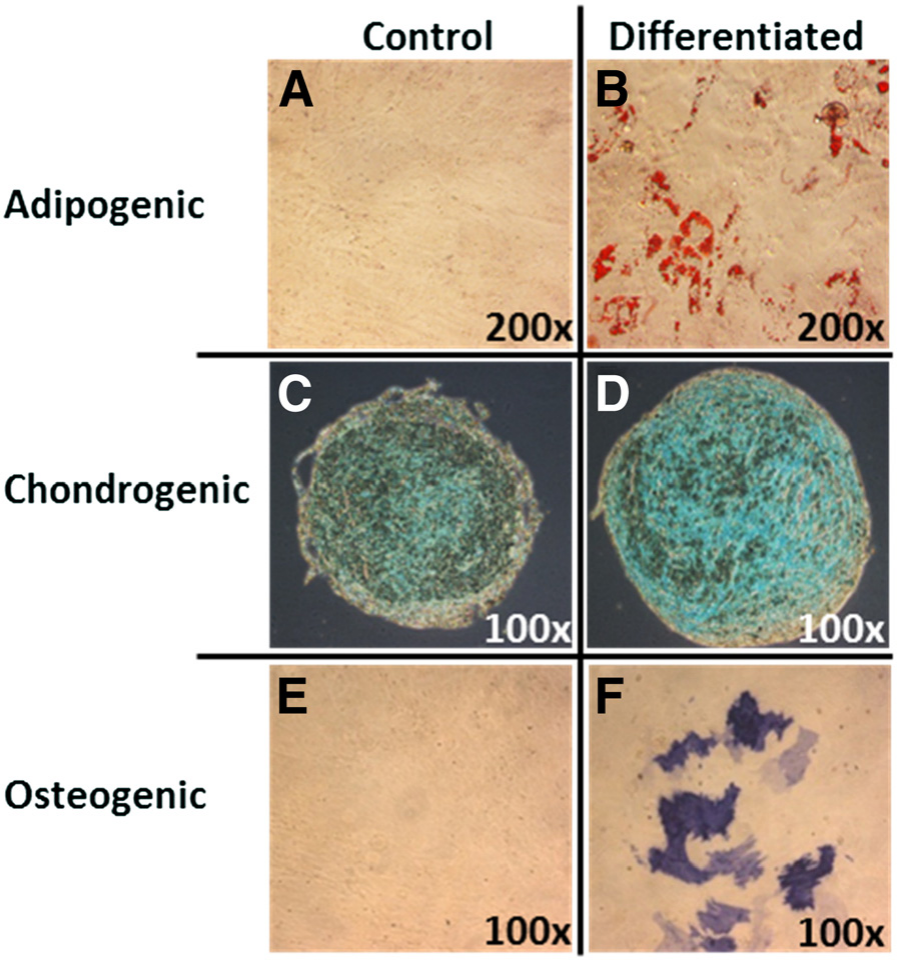
**Tri-lineage differentiation potential of UCX® cells.** Multi-lineage differentiation potential of UCX® cells were qualitatively analyzed by histological staining methods. Adipogenic differentiation was assessed with Oil Red O staining both in (**A**) control and (**B**) differentiated samples; chondrogenic differentiation with Alcian Blue staining in (**C**) control and (**D**) differentiated samples; and osteogenic differentiation with Alkaline Phosphatase staining in (**E**) control and (**D**) differentiated samples. The photos are representative of one out of eight umbilical cords tested.

UCX® cells were expanded to P5 and incubated with specific differentiation media as described in the methods. Results showed that UCX® cells have the capacity for tri-lineage differentiation into adipocytes, chondrocytes and osteoblasts (Figure 2A-F). Control samples on the left column are cell cultures undergoing same culture conditions and specific staining reactions but without addition of differentiation factors. For chondrogenic differentiation, the natural tendency for UCX® cells to form three-dimensional aggregates has become noticeable, even without the addition of differentiaion factors (Figure 2C). In any case, chondrospheres resulting from terminal chondrogenic differentiation are consistently larger, more regularly shaped, and strongly stain positive for alcian blue (Figure 2D).

#### UCX® cells have the capacity to suppress T-cell proliferation and to induce treg conversion

**Figure 3:**
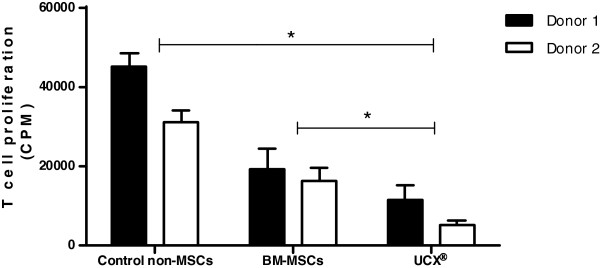
**UCX® cells suppress T-cell proliferation.** In a MLR, suppression of T-cell proliferation was analyzed in the presence of UCX® cells, BM-MSCs and control non-MSCs (Molt-4) following activation of PBMCs from two different donors with anti-CD3, anti-CD28 and IL-2. Data is presented as mean ± SEM (n = 4) and statistically significant differences are indicated with asterisks (non-parametric test Mann Whitney, *P < 0.05).

Wharton’s jelly-derived MSCs have been shown to be able to be safely used in allogeneic applications due to both their lack of immunogenicity when compared to other MSCs, and their marked capacity for localized immunosuppression. In order to evaluate the capacity of UCX® cells to modulate T-cell activation, peripheral blood mononuclear cells (PBMCs) from 2 different donors were stimulated with anti-CD3, anti-CD28 and IL-2 while co-cultured with irradiated UCX® cells, bone marrow-derived mesenchymal stem cells (BM-MSCs) and tumor cells belonging to an acute lymphoblastic leukemia adult cell line (Molt4) as non-MSC control. Results showed that in two different donors, both MSC-type cells have an immunosuppressive effect when compared to Molt-4 (Figure 3). Moreover, UCX® cells were able to inhibit T-cell proliferation more significantly than BM-MSCs, suggesting that these cells are more immunosuppressive than BM-MSCs.

The capacity of UCX® cells to take a multi modal approach to immune regulation, through induction of Tregs was also evaluated. Tregs are a sub-set of naive CD4+CD25+ T-cells that express the Foxp3 transcription factor and become regulatory in the periphery in response to a variety of signals, including antigen exposure in the presence of immunosuppressive cytokines such as TGF-β [28]. Previous studies have demonstrated that not all CD4+CD25+ cells concomitantly expressed Foxp3, while only very few Foxp3+ cells resided in the CD25- population [16,29]. However, Foxp3 remains the best marker to identify regulatory T-cell populations [30]. Therefore, in this study we assessed Foxp3 expression in FACS-sorted CD4+ T-cells as indication of Treg conversion.

In order to address the putative effect of UCX® cells in inducing the conversion of Treg cells we used an in vitro co-culture system where sorted polyclonal populations of CD4+CD25- T-cells from human donors were activated in the presence or absence of UCX® cells. It has been shown that immunosuppressive reagents can induce Treg cells independently of the addition of exogenous TGF-β to the cultures [31]. The therapeutic induction of Foxp3+ Treg cells can then protect from inflammatory pathology such as arthritis or neuro-inflammation [32,33].

**Figure 4:**
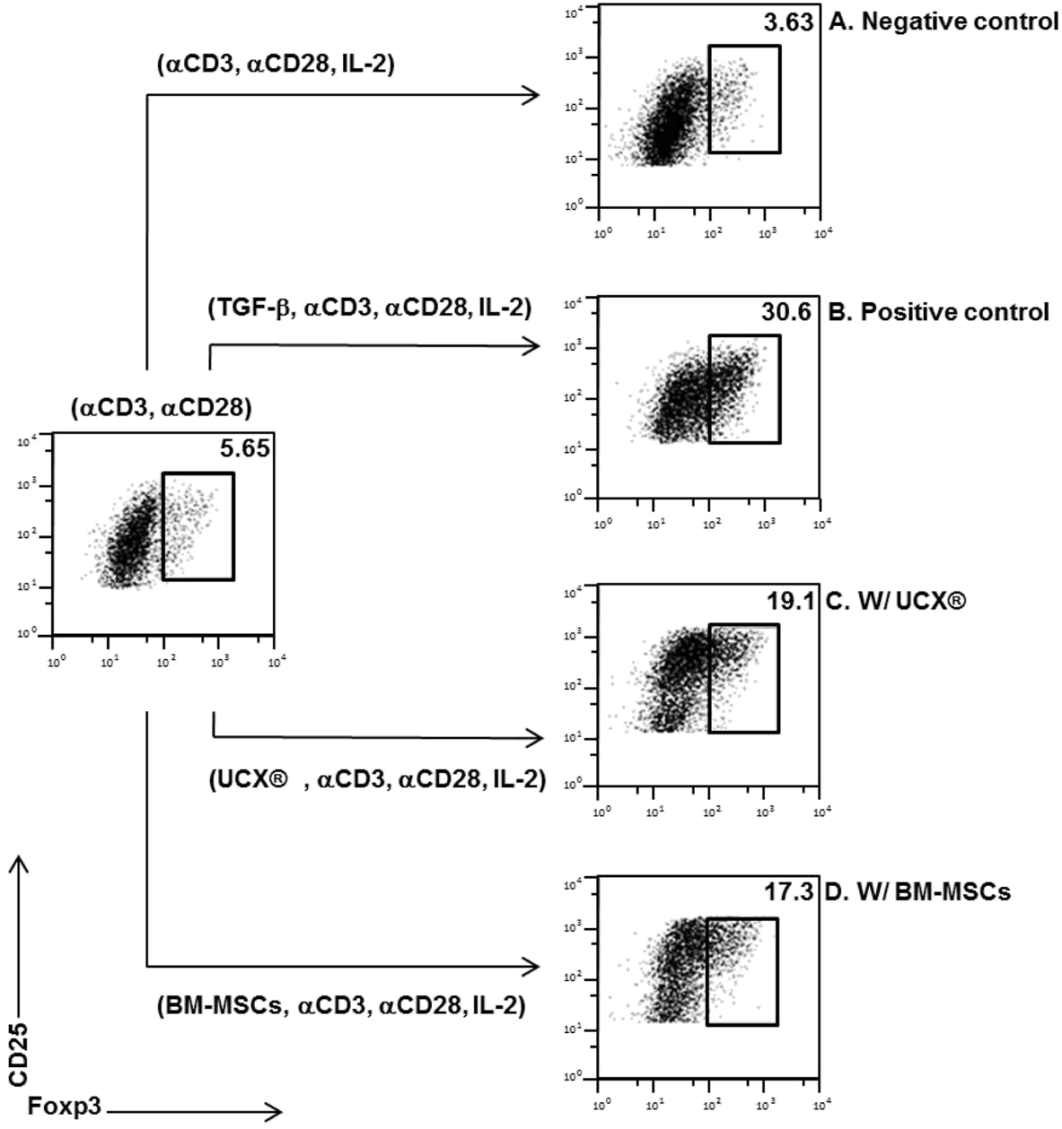
**UCX cells induce T-cell conversion into Treg cells.** FACS-sorted CD3+ CD4+ CD25- T-cells were activated with anti-CD3, anti-CD28 and IL-2 alone (**A**) and with the addition of TGF-β (**B**). The effect of irradiated UCX® cells (**C**) and BM-MSCs (**D**) in T-cell conversion was analyzed in cultures without exogenous TGF-β by flow cytometry analysis of CD25 and Foxp3 expression.

We found, as expected, that T-cell activation with anti-CD3, anti-CD28, and IL-2 in the absence of exogenous TGF-β did not lead to induction of CD4+Foxp3+ cells (Figure 4A - negative control). On the contrary, we confirmed that addition of TGF-β to the culture resulted in considerable Treg conversion (positive control, Figure 4B). In the experimental group we found that the presence of UCX® cells resulted in significant induction of Treg conversion of over 18% of the CD4+ cell population (Figure 4C). Although not as efficient as UCX® cells, BM-MSCs also promoted conversion of Tregs (approx. 16%, Figure 4D). Taken together, these data establish the ability of UCX® cells to induce Foxp3+ Treg cells, as well as a potent effect in suppressing T-cell activation.

#### UCX® cells have the capacity to reduce inflammation in vivo in an acute carrageenan-induced arthritis (CarrIA) model

**Figure 5:**
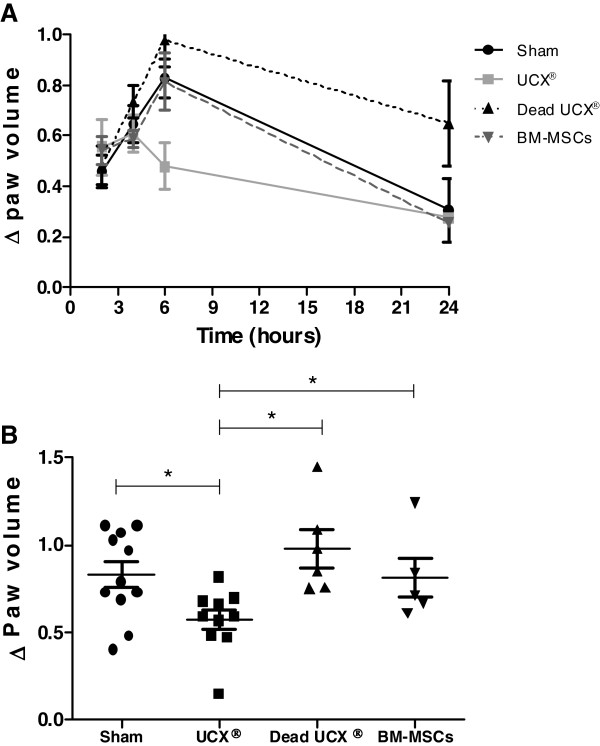
**UCX® cells suppress carragenan-induced arthritis.** UCX® arthritic anti-inflammatory activity in vivo was assessed by measuring the (**A**) paw volume at several time points both before and after carrageenan induction relative to the pre-injection volume and (**B**) paw volume at maximum peak time - 6 h, relative to the pre-injection volume. Data is presented as mean ± SEM and statistically significant differences are indicated with asterisks (non-parametric test Mann Whitney, *P < 0.05).

In order to assess if UCX® cell immunosuppression properties could result in arthritic anti-inflammatory activity in vivo, cells were administered in an acute carrageenan-induced arthritis (CarrIA) footpad edema model. The CarrIA is a model for acute inflammation and provides a rapid assessment of the anti-inflammatory effect of a certain compound. In this study, different groups (n = 12 for Sham and UCX®, n = 6 for dead UCX® and BM-MSCs) of 7 to 8 week-old Wistar rats were treated either with PBS vehicle (Sham control), live viable UCX® cells (dose 1.7x10^6^ cells), dead non-viable human UCX® cells (dose 1.7 x10^6^ cells), and human BM-MSCs (dose 1.7x10^6^ cells) 1 hour prior to challenge with λ-carrageenan in the right hind footpad. Edema was measured as the increase in paw volume (milliliters) after carrageenan injection (relative to the pre-injection volume). Results showed that in vehicle-treated paws (sham control) footpad volume peaked at time 6 h and regressed back to near baseline levels 24 h after carrageenan induction (Figure 5A). Figure 5A also shows that non-viable UCX® cells induced a slightly increased inflammatory response up to 6 h when compared to the Sham control. This inflammatory response was not observed with either viable BM-MSCs or UCX® cells. Nevertheless, while the injection with BM-MSCs caused a similar effect as Sham control, injection of UCX® cells strongly attenuated paw inflammation, clearly reverting edema formation in a cell-specific fashion. In agreement with the immunosuppression activity observed in vitro (Figures 3 and 4), Figure 5B showed a statistically significant reduction (over 30%) in hind paw inflammation at time 6 h in UCX® -treated animals when compared to Sham control and BM-MSCs-treated animals. In addition, the anti-inflammatory effect of UCX® cells observed in vivo was dependent on cell viability, as seen by the loss of activity observed when using non-viable UCX® cells. These results showed that in addition to the observed immunosuppressive activity seen in vitro, UCX® cells have an anti-inflammatory effect in vivo.

#### UCX® treatment prevented the development of arthritic inflammation manifestations in a chronic adjuvant-induced arthritis (AIA) model

**Figure 6:**
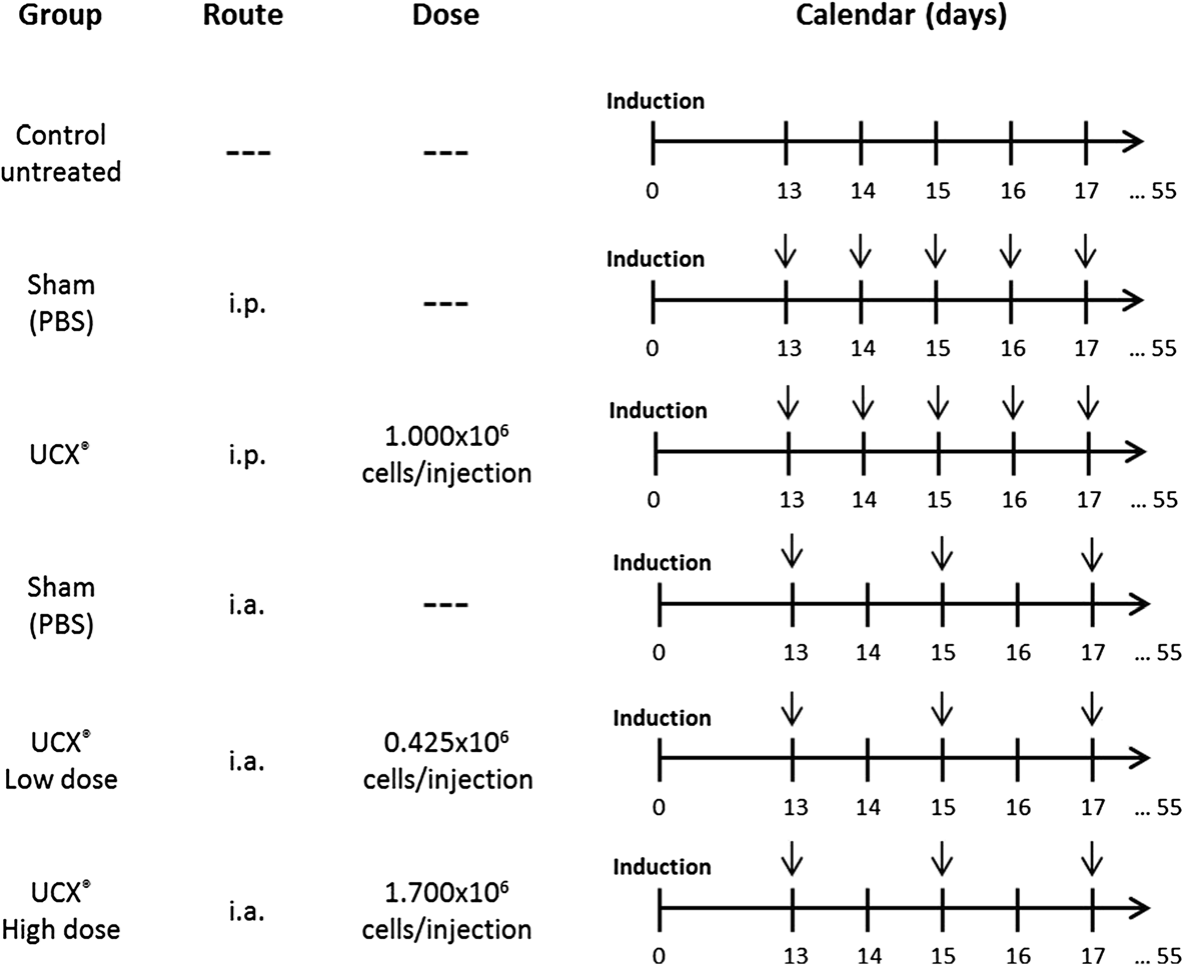
**Treatment schedule and UCX® dosage for the chronic adjuvant induced arthritis (AIA) model.** In the AIA experiment, animals were randomly allocated to 6 different treatment groups. One group of induced animals remained untreated, being referred as Control untreated. Other 2 groups of induced animals received PBS by intra articular (i.a.) or intra peritoneal (i.p.) routes, respectively, being referred as Sham groups. A total of five i.p. administrations (dose: 1.000x10^6^ cells/injection) were performed every day, from day 13 to 17. In turn, three i.a. administrations (either low dose: 0.425x10^6^ cells/injection; or high dose: 1.7x10^6^ cells/injection) were performed every other day, for five days, from day 13 to 17 after arthritis induction. Arrows indicate injection days. The experiment lasted for 55 days.

The immunoregulatory effects of UCX® on T-cells, as well as the anti-inflammatory activity observed in the xenogeneic CarrIA model prompted to investigate the potential therapeutic effects of UCX® cells in the chronic AIA model, an arthritis model that shares many clinical and immunological features with RA. Both local (intra- articular- i.a.) and systemic (intra-peritoneal – i.p.) routes of administration were tested and, in the case of i.a., a dose effect for UCX® cells was also evaluated. The treatment schedule and UCX® dosage are shown in Figure 6. Arthritis was induced in the sub-plantar area of right hind paws and the onset of arthritic manifestations was observed at day 13, when arthritis became established (AI score of 4, see methods section).

**Figure 7:**
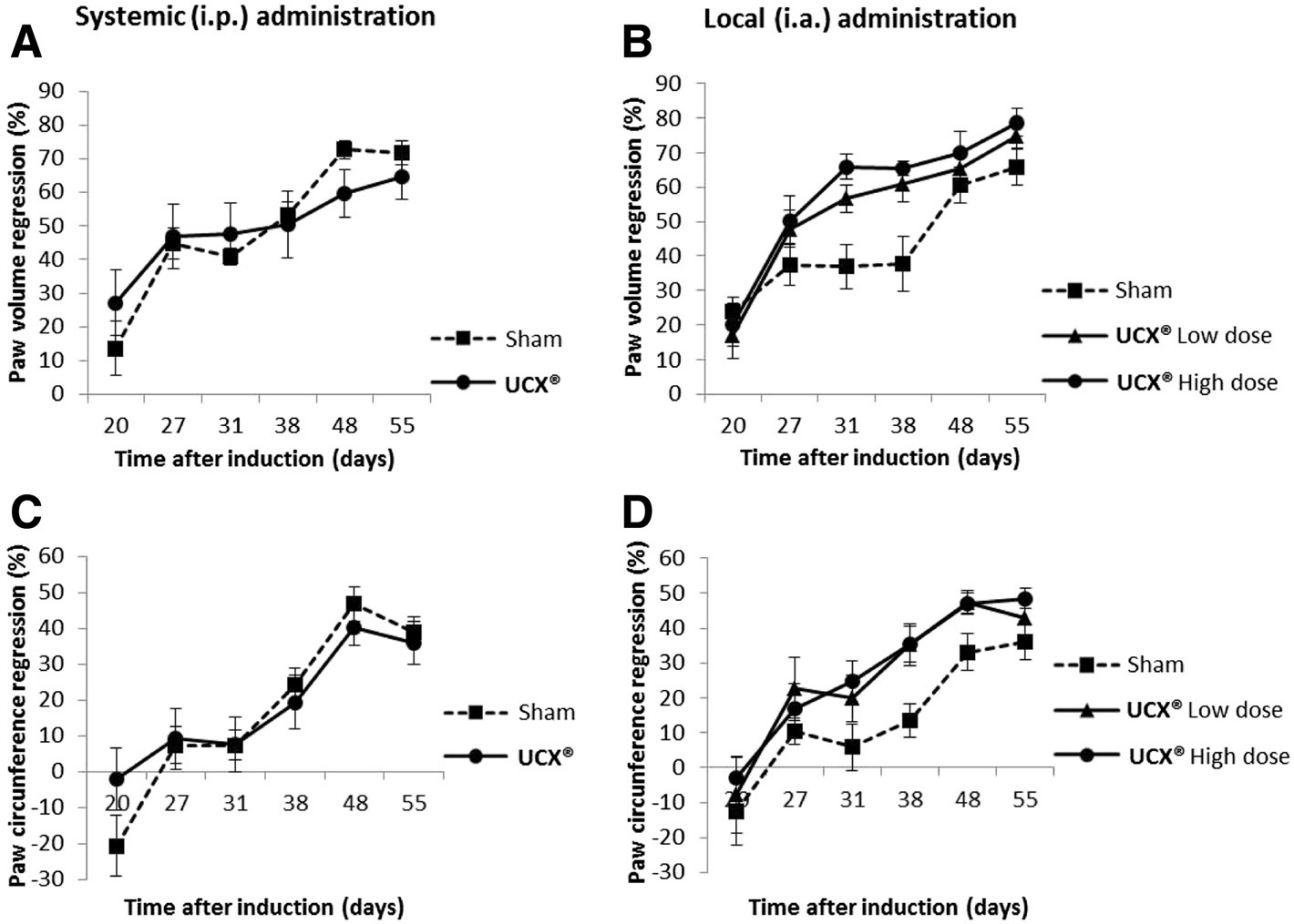
**Differential local effects of (i.p.) and (i.a.) UCX® cell administration in the AIA model.** Graphical representations of % variation analysis of (**A,B**) paw volume regression related to naive animals, and (**C, D**) paw circumference regression related to naive animals, during the time scoped by the AIA model experiment, both in (**A,C**) systemic and (**B, D**) local administration of UCX® cells and Sham controls. Data is presented as mean ± SEM (n = 8).

Figure 7 (A-D) depicts % variations (regression) related to naive animals in both, volume of posterior right hind paw and ankle circumference, for systemic (i.p.) and local (i.a.) administration groups. In all experimental groups, animals typically lost near to 20% body weight during the acute phase of the disease, between days 13 and 20 after induction (results not shown). Body weight recovery between days 20 and 55 was accompanied by regression of hind paw inflammation in all experimental groups. However, UCX® i.a. administration proved to be highly effective in ameliorating local inflammatory signals when compared to UCX® i.p. administration. While UCX® i.p. administration has shown no improvements in local inflammation, when compared to the Sham control (Figure 7A and 7C), UCX® i.a. administration promoted a significantly faster regression of both hind paw volume and hind paw circumference, from days 20 to 55 (Figure 7B and 7D). The promotion of hind paw volume regression caused by UCX® cells was shown to be dose dependent, with the highest UCX® cell dose reaching an average of nearly 80% volume recovery at day 55 (Figure 7B).

**Figure 8:**
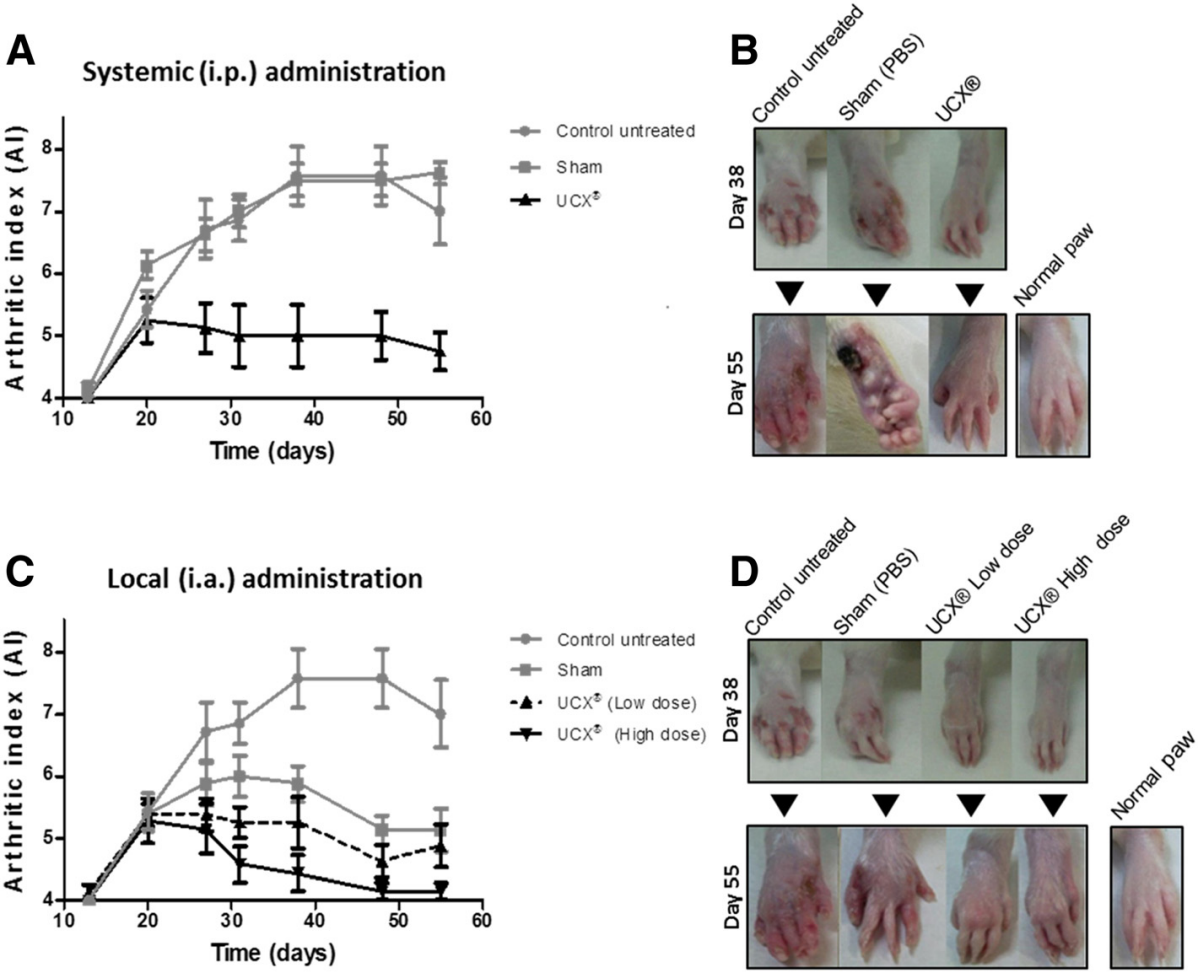
**Local and systemic delivery of UCX® cells prevent global arthritic manifestations in the AIA model.** Graphical representations of (**A, C**) AI variation during the time scoped by the AIA model experiment. Animals were scored at pre-selected times based on the sum of the following grades: 0 = normal paw; 1 = for each inflamed paw; 1 = tail lesion; 1 = joint rigidity or deformity; 1 = wounded paw; 1 = infected paw; 1 = necrotic paw. The sum of the parameters was calculated to a maximum AI of 9. Data is presented as mean ± SEM (n = 8). The evolution of clinical signs was photographically recorded (**B, D**) and compared to both normal and Sham-treated animals. Photos are representative of 8 animals.

When all local and systemic parameters were taken together, as a measure of the arthritic index (AI), the positive effect of UCX® treatment became clear. As it can be seen in Figure 8A and 8C the severity of AIA was rapidly attenuated overtime in UCX®-treated mice, as compared with both control untreated and vehicle (Sham) treated groups. As observed in Figure 8A, the Sham group reached a maximum AI score of 7.6 (day 38) while i.p. UCX® -treated animals reached a maximum of 5 after arthritis induction (day 21). In the case of local treatment (Figure 8C), the Sham group reached a maximum AI of 6 at day 32 after induction and significant lower values were obtained for treated groups, in a dose-dependent fashion. The Sham group presented a decrease in AI values following the same trend as treated groups but with higher severity index (Figure 8C). This can possibly be interpreted as either a local wash-away effect, reducing the infection locally, or a decompressing effect due to i.a. injection that released pus, thereby reducing hind paw volume. Just like the promotion of volume and paw circumference regression before (Figure 7D and 7F), the reduction of AI scores during the follow up of i.a. administration was shown to be UCX® dose-dependent (Figure 8C). The AI scores were representative of the relative AIA severity and were clearly supported by macroscopic observations. Unlike UCX® treated animals, control untreated and Sham group animals have evolved from an hind-paw inflammation stage to the presence of articular deformities, low mobility, tail and paw lesions by day 38; and ultimately developed clear signs of paw necrosis and irreversible joint rigidity between days 48 and 55 (Figure 8B and 8D). In turn, animals in both i.p. and i.a. UCX®-treated groups have reached a mild articular inflammation stage, with subtle-to-mild systemic signs of disease up to day 38. From day 38 to 55, animals treated systemically, through an i.p. mode of administration, have recovered in part the local arthritic symptom, suggesting that UCX® administered systemically affect the clinical course of AIA. In turn, animals treated locally, through an i.a. mode of administration, with a high UCX® dose, presented apparent full recovery of both local and systemic manifestations, as well as local motor hindrance (Figure 8B and 8D). The results suggest that UCX® cells were successful in protecting against the development of more severe arthritic manifestations, from a period with already observable manifestations, day 13 (AI of 4) to day 38 after induction Additional to this protective effect, both i.p. and i.a.-administered UCX® cells were able to promote regression of arthritic signs, significantly reducing the AI parameters. UCX® cells can thus be an effective and promising new approach for treating both local and systemic arthritis.

## Discussion

The present study shows that human UCX® cells are a homogenous population of stem cells that comply with the current definition of mesenchymal stromal cell (MSC) as established by the International Society for Cellular Therapy (ISCT) [34] namely, cells adhere to a plastic surface, at least 95% of UCX® cells in the population are consistently positive for the cell surface markers CD44, CD73, CD90 and CD105 and less than 2% positive for CD14, CD19, CD31, CD34, CD45 and HLA-DR, and cells are capable of undergoing tri-lineage differentiation into adipocytes, chondrocytes and osteoblasts. More importantly, UCX® cells have the capacity to inhibit human T-cell proliferation, and concomitantly induce the conversion of T-cells to the Treg sub-type of regulatory cells. Our results showed that such capacity is more evident in UCX® cells than in BM-MSCs, sometimes considered the gold standard for MSC therapy applications.

Our results also showed that, in a xenogeneic setting, human UCX® cell immunosuppression properties can result in arthritic anti-inflammatory activity in vivo*,* both in acute and chronic arthritis animal models. UCX® cells administered in a rat CarrIA model strongly attenuated hind paw inflammation, protecting against acute edema formation in a cell-specific fashion. Furthermore, UCX® action in vivo was dependent on cell viability. The anti-inflammatory effect was not observed when using human BM-MSCs in the same acute model, in accordance to the lower immunosuppressive capacity observed in vitro. The differences in anti-inflammatory behavior between UCX® cells and BM-MSCs, as seen in a CarrIA model, agree with previous studies where UC-MSCs have proven effective in treating collagen-induced arthritis in mice [16], while BM-MSCs have been shown to be ineffective in AIA rats [35]. These different observations also show that MSCs from different tissue sources should not be treated as equal in terms of their immunosuppressive and anti-inflammatory activities and should therefore be discussed and evaluated as separate treatment strategies.

In fact, a comparative study between BM-MSCs and Wharton’s Jelly-derived UC-MSCs, primed with key pro-inflammatory cytokines IFN-γ and TNF-α, showed that the extent of immunosuppression was more accentuated with UC-MSCs than with BM-MSCs in both cases [14]. Furthermore, priming BM-MSCs with IFNγ enhanced suppression of mitogen-driven lymphocyte proliferation only, whereas IFN-γ primed UC-MSCs were better suppressors in MLRs, in agreement with our observations using UCX® cells. Additionally, PHA-stimulated lymphocytes in co-cultures of primed/unprimed UC-MSCs and BM-MSCs showed that UC-MSCs resulted in an early activation of a negative co-stimulatory molecule, CTLA4, which was not evident with BM-MSCs [14]. In summary, different expression patterns indicated that UC-MSCs were less immunogenic, more immunosuppressive and more inflammatory than BM-MSCs [14].

More recently, the observations described above have been further consubstantiated. High throughput gene expression analysis using microarrays (GeneShip® from Affymetrix) have shown that UCX® cells express significantly higher levels of IL-8, IL1β, LIF and TGFβ when compared with BM-MSCs. The receptors for pro-inflammatory cytokines such as IFN-γ, TNFα and IL-1β were also found substantially expressed in UCX® cells, which is consistent with the reported ability of these cells to home to inflammation sites and become immunosuppressive in response to inflammatory signals (results to be published elsewhere). The expression of LIF and TGF-β are particularly relevant for this study since both have been directly implicated in Treg induction [36,37].

Finally, the combined immunosuppressive and anti-inflammatory capabilities of human UCX® cells were successfully applied to the treatment of AIA in vivo. treated animals, through an i.a. mode of administration, presented full recovery of both local and systemic AIA signs, as well as local motor hindrance. Overall, the results suggest that UCX® cells have been successful in protecting against the development of more severe manifestations of arthritis, from a period with already observable manifestations at day 13 (AI of 4) up to day 38 after induction, when Sham controls and untreated animals developed clearly more severe conditions. Additional to this protective effect, both i.p. and i.a.- administered UCX® cells were able to promote regression of arthritic manifestations, reducing all measurable and observable IA parameters. To our knowledge, this study presents the first evidence of successful treatment of chronic adjuvant-induced arthritis (AIA) where MSC-type cells have been administered after the appearance of clear AIA manifestations.

Human UC-MSCs have been shown previously to suppress the various inflammatory effects of FLSs and T-cells of RA in vitro, and to attenuate the development of collagen-induced arthritis (CIA) in mice. In addition, the immunosuppressive activity of UC-MSCs was prolonged by the participation of Tregs [16]. Tregs modulate a variety of immune functions from initial T-cell and B-cell activation to effector function in the target tissue, and appear to play a critical role in the maintenance of self-immune tolerance in RA [28,38]. Some authors consider that CD4+CD25+ cells in PBMCs contain a mixture of both thymic natural Tregs (nTregs) and periphery induced Tregs (iTregs). The generation of iTregs is dependent upon the presence of both TGF-β and TGF-β receptor signals. Furthermore, iTregs are known to be superior to nTregs in ameliorating established CIA, as well as other types of ongoing autoimmunities [39]. We would like to propose that the use of UCX® cells is an effective and promising new approach for treating autoimmune-derived inflammatory arthritis symptoms by a mechanism involving homing to inflammation sites and immunosuppression via a two-way pathway: 1- repression of T-cell proliferation and 2- TGF-β-dependent paracrine promotion of iTreg conversion. The beneficial effects can be extended to the systemic nature of autoimmune inflammatory arthritis disorders, such as RA.

## Conclusion

The present study has provided strong evidence that UCX® cells are strong candidates to become an Advanced Therapy Medicinal Product (ATMP) for the treatment of inflammatory arthritis in the near future.

## Competing interests

The authors declare that they have no competing interests.

## Authors’ contributions

JMS conceived the overall design of the study, coordinated the study and drafted the manuscript. RB has made substantial contributions to conception and design of the study, analysis and interpretation of data; and helped drafting the manuscript. SIS, MMG and SC have carried out the CarrIA and AIA model experiments. AAD and SCPA have carried out the MLR assays. JA, MF, MT and JPM have performed UCX® cell isolation, expansion, differentiations and the other characterization studies using flow cytometry. LG, MEMC and PC have revised the manuscript critically and have given scientific input. HC has contributed to the conception and design of the study and has revised the manuscript critically for important intellectual content. All authors have read and approved the final manuscript.
